# Probiotic *Lactobacillus rhamnosus* GG (LGG) restrains the angiogenic potential of colorectal carcinoma cells by activating a proresolving program via formyl peptide receptor 1

**DOI:** 10.1002/1878-0261.13280

**Published:** 2022-07-20

**Authors:** Federica Liotti, Maria Marotta, Daniela Sorriento, Chiara Pagliuca, Valeria Caturano, Giuseppe Mantova, Elena Scaglione, Paola Salvatore, Rosa Marina Melillo, Nella Prevete

**Affiliations:** ^1^ Department of Molecular Medicine and Medical Biotechnology University of Naples Federico II Italy; ^2^ Institute of Experimental Endocrinology and Oncology (IEOS) CNR Naples Italy; ^3^ Department of Advanced Biomedical Sciences University of Naples Federico II Italy; ^4^ Department of Chemical, Materials and Production Engineering University of Naples Federico II Italy; ^5^ CEINGE, Biotecnologie Avanzate s.c.ar.l. Naples Italy; ^6^ Task Force on Microbiome Studies University of Naples Federico II Italy; ^7^ Department of Translational Medical Sciences University of Naples Federico II Italy

**Keywords:** angiogenesis, colon cancer, formyl peptide receptor 1, *Lactobacillus rhamnosus* GG, LGG, specialized proresolving mediators

## Abstract

Formyl peptide receptors (FPR1, FPR2 and FPR3) are innate immune sensors of pathogen and commensal bacteria and have a role in colonic mucosa homeostasis. We identified FPR1 as a tumour suppressor in gastric cancer cells due to its ability to sustain an inflammation resolution response with antiangiogenic potential. Here, we investigate whether FPR1 exerts similar functions in colorectal carcinoma (CRC) cells. Since it has been shown that the commensal bacterium *Lactobacillus rhamnosus* GG (LGG) can promote intestinal epithelial homeostasis through FPR1, we explored the possibility that it could induce proresolving and antiangiogenic effects in CRC cells. We demonstrated that pharmacologic inhibition or genetic deletion of FPR1 in CRC cells caused a reduction of proresolving mediators and a consequent upregulation of angiogenic factors. The activation of FPR1 mediates opposite effects. Proresolving, antiangiogenic and homeostatic functions were also observed upon treatment of CRC cells with supernatant of LGG culture, but not of other lactic acid or nonprobiotic bacteria (i.e. *Bifidobacterium bifidum* or *Escherichia coli*). These activities of LGG are dependent on *FPR1* expression and on the subsequent MAPK signalling activation. Thus, the innate immune receptor FPR1 could be a regulator of the balance between microbiota, inflammation and cancer in CRC models.

## Graphical abstract

The commensal probiotic bacteria *Lactobacillus rhamnosus* GG (LGG), by activating the innate immune receptor formyl peptide receptor 1 (FPR1), influence normal intestinal epithelial homeostasis and sustain wound restitution. We demonstrated that LGG, but not other lactic acid or nonprobiotic bacteria, activate an inflammation resolution program that dampens the angiogenic response of colorectal carcinoma cells.

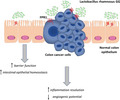

**Abbreviations**
AA: arachidonic acid
Abs: antibodies
Akt: protein kinase B
ALOX15: arachidonate 15‐lipoxygenase
ALOX15B: arachidonate 15B‐lipoxygenase
ALOX5: arachidonate 5‐lipoxygenase
Ang1: angiopoietin 1
AnxA1: annexin A1
*B. bifidum*
: *Bifidobacterium bifidum*
BLT‐1: block lipid transport‐1
cfu: colony‐forming unit
ChemR23: chemerin receptor 23
CM: conditioned media
CRC: colorectal carcinoma
CsH: cyclosporine
CXCL1: chemokine (C‐X‐C motif) ligand 1
DHA: docosahexaenoic acid
DPP IV: dipeptidyl peptidase‐IV
*E. coli*
: *Escherichia coli*
ECM: extracellular matrix
ECs: endothelial cells
EG‐VEGF: human endocrine gland‐derived vascular endothelial growth factor
EIA: enzyme immunoassay
ELISA: enzyme‐linked immunosorbent assay
EPA: eicosapentaenoic acid
FACS: fluorescence‐activated cell sorting
fMLF: N‐formyl‐Met‐Leu‐Phe
FPR: formyl peptide receptor
FPR1: formyl peptide receptor 1
FPR2: formyl peptide receptor 2
GC: gastric cancer
GDNF: glial cell line‐derived neurotrophic factor
GPR32: G protein‐coupled receptor 32
HUVECs: human umbilical vein endothelial cells
IBD: inflammatory bowel disease
IGFBP‐1: insulin‐like growth factor‐binding protein 1
IGFBP‐2: insulin‐like growth factor‐binding protein 2
LGG: *Lactobacillus rhamnosus*
LTB_4_
: leukotriene B4
LXA_4_
: lipoxin A_4_
LXB_4_
: lipoxin B_4_
MAPK: mitogen‐activated protein kinase
MaR: maresins
MCP‐1: monocyte chemoattractant protein‐1
MMP8: matrix metallopeptidase 8
MMP9: matrix metallopeptidase 9
mRNA: messenger RNA
MRS: sharpe broth medium
NSCLC: non‐small‐cell lung cancer
PCR: polymerase chain reaction
PD: protectins
PD‐ECGF: platelet‐derived endothelial cell growth factor
PDGF‐AA: platelet‐derived growth factor AA
PDGF‐AB: platelet‐derived growth factor AB
PDGF‐BB: platelet‐derived growth factor BB
PGE_2_
: prostaglandin E_2_
PIGF: placental growth factor
PRRs: pattern recognition receptors
PUFA: polyunsaturated fatty acid
RNA: ribonucleic acid
RvD1: resolvin D1
RvDs: resolvins D
RvEs: resolvins E
SD: standard deviation
shCTR: nontargeting short hairpin
shFPR1: short hairpin targeting FPR1
shRNA: short hairpin RNA
SN: supernatant
SPMs: specialized proresolving mediators
STAT3: signal transducer and activator of transcription 3
TGF‐beta1: transforming growth factor‐beta 1
TIMP‐1: tissue inhibitor matrix metalloproteinase 1
TIMP‐4: tissue inhibitor matrix metalloproteinase 4
TLR7: toll‐like receptor 7
TS: Tryptic Soy
uPA: urokinase plasminogen activator plasma
VEGF‐A: vascular endothelial growth factor A
VEGF‐B: vascular endothelial growth factor B
VEGF‐C: vascular endothelial growth factor C
VEGF‐D: vascular endothelial growth factor D
ω‐3: omega‐3
ω‐6: omega‐6

## 1 Introduction

Chronic inflammation is a risk factor for colorectal carcinoma (CRC) onset [1], as strongly suggested by the increased predisposition to colon carcinogenesis of inflammatory bowel disease (IBD) patients [2].

In recent years, unresolved chronic inflammation has been associated with insufficient production of mediators, which are actively involved in the resolution of inflammation [3, 4]. Molecules diverse in nature are able to actively participate in different moments of the resolution program [3]: (i) lipidic autacoids known as specialized proresolving mediators (SPMs) [4]; (ii) proteic mediators [e.g. annexin A1 (AnxA1), adrenocorticotropic hormone, chemerin peptides, and galectin‐1] [3, 5]; (iii) gaseous mediators (nitric oxide, hydrogen sulfide and carbon monoxide) [3]; (iv) the adenosine, a purine nucleoside generated by the dephosphorylation of adenine [6]; and (v) neuromodulators such as acetylcholine and other neuropeptides produced by vagus nerves [7].

Specialized proresolving mediators are lipidic mediators derived from omega‐6 (ω‐6) arachidonic (AA), or ω‐3 eicosapentaenoic (EPA) and docosahexaenoic (DHA) essential polyunsaturated fatty acids (PUFA) through the activity of lipoxygenases 5 and 15 (ALOX5/15). The best‐characterized SPMs are lipoxins (LXA_4_, LXB_4_), E‐ and D‐series resolvins (RvEs and RvDs), protectins (PD) and maresins (MaR) [8]. They exert anti‐inflammatory, antiangiogenic and proresolving effects subsequent to inflammatory conditions [4, 8, 9].

We recently described a novel function of SPMs in gastric cancer (GC) demonstrating that RvD1 and LXB_4_ suppress angiogenesis, thus inhibiting tumour growth. We also demonstrated that formyl peptide receptor 1 (FPR1), a member of the FPR family, controls SPM production in GC [9, 10], functioning as a tumour suppressor [11, 12]. FPRs are pattern recognition receptors (PRRs) known to balance inflammatory and proresolving responses by sensing host‐derived and bacterial products [13, 14].

Several reports point to a crucial protective role of proresolving pathways also in CRC carcinogenesis [15]. Indeed, it has been demonstrated that CRC is associated with a reduced intake of ω‐3 PUFA [16] and that dietary supplementation of ω‐3 PUFA exerts anticancer effects in CRC [17]. Furthermore, *ALOX15* has been described as a tumour suppressor in CRC [18] and specific SPMs (i.e. RvD1, LXA_4_) have been demonstrated to exert antitumour activity in CRC models [19, 20, 21, 22, 23, 24].

Intestinal inflammatory conditions are strongly influenced and in turn affect microbiota composition [1, 25]. In the last years, several studies in CRC patients and experimental models demonstrated that colon tumorigenesis is associated with significant alterations of intestinal microbial composition termed as dysbiosis [26, 27]: in CRC patients, specific bacterial species are over‐represented compared with those in noncancerous patients and exert protumorigenic function(s), while other species are under‐represented and exert tumour suppressive functions [26, 27, 28].

*Lactobacillus rhamnosus* GG (LGG) is a commensal bacterium used as probiotic in order to reverse intestinal dysbiosis [29]. Several preclinical studies point to its effects in reducing chronic inflammation linked to CRC development [30]: LGG has been demonstrated to regulate homeostasis and restitution following colonic wounds in mice [31, 32, 33, 34]; in CRC models, LGG activates proapoptotic and antimetastatic responses [35, 36], lowers inflammation and favours adaptive protective immune responses against cancer cells [37]. It has been shown that LGG activity in intestinal epithelial cells depends on the expression of FPR1 [32].

Since the gastrointestinal tract is continuously exposed to external insults, proresolving pathways are particularly important to balance inflammatory responses for its homeostasis [4]. Thus, we investigated the role of FPR1 in CRC cells in order to verify the possibility that it functions as a regulator of inflammation resolution, angiogenesis and cancer. Moreover, we evaluated whether homeostatic and anticancer effects of LGG in CRC models could depend on its ability to activate a proresolving response. In particular, we investigated the possibility that LGG could activate a proresolving and an antiangiogenic response in CRC cells by stimulating FPR1.

Our data confirm this hypothesis and highlight the possibility that FPR1 could be exploited in order to increase the proresolving and inhibit the angiogenic potential of CRC cells, also through the use of probiotics.

## 2 Materials and methods

### 2.1 Cell culture

The HCT116 and HT29 cell lines derived from colorectal carcinoma (CRC) were kindly provided by S. Scala (Istituto Nazionale Tumori‐IRCCS‐Fondazione G. Pascale, Napoli, Italy) and grown as elsewhere described [38]. To generate HCT116 cells stably expressing *FPR1* shRNA, we used pools of 5 constructs (Qiagen, Valencia, CA, USA) containing 21‐mer short hairpin RNAs (shRNA) directed to various coding regions of the target gene. Transfectants were selected in medium with 500 ng·mL^−1^ puromycin [10]. Human umbilical vein endothelial cells (HUVECs) from Cell Biologics (Chicago, IL, USA) were grown in human endothelial cell medium with the addition of VEGF, heparin, EGF, FGF, hydrocortisone, L‐glutamine, antibiotic/antimycotic solution and FBS according to the manufacturer's instructions (Cell Biologics) [39].

Cell treatments to verify (a) mRNA changes, (b) enzyme or receptor protein expression, (c) AnxA1 expression, (d) signalling pathway activation and (e) autacoid release were made in serum‐free media and after a 12‐h serum starvation. Instead, cell treatment for VEGF‐A release and to collect cell culture supernatants to be used in capillary formation assay was performed in 1% FBS to improve the stability of VEGF‐A. The same conditions were used when bacterial supernatants (SN) were used to treat CRC cells; the relative control sample of each bacterial strain SN was the same titration of the culture broth.

Treatments of CRC cells were made with fMLF at 1 nm, the concentration at which it binds specifically to FPR1 [13]. The SPMs were used again at 1 nm, the same optimal concentration used to inhibit angiogenesis in the GC model [10].

The functional involvement of GPR32 or MAPK signalling in CRC proresolving responses was evaluated by preincubating cells for 30 min with an anti‐GPR32 neutralizing antibody (Ab) (1 μg·mL^−1^) (GeneTex, Irvine, CA, USA) or the MAPK signalling inhibitor U0 126 (10 μm) (Cell Signaling, Danvers, MA, USA), respectively, before proceeding with the specific treatment.

### 2.2 Bacterial culture and supernatant preparation

The bacterial strains used in this study were as follows: *Lactobacillus rhamnosus* (Hansen) Collins *et al*. (LGG) (ATCC 53103), obtained from the ATCC (Manassas, VA); *Escherichia coli* ATCC 13762 (*E. coli*), used as control of nonprobiotic bacteria; and *Bifidobacterium bifidum* (B. bifidum), an anaerobic lactic acid bacterium isolated from the ProBiota Bifido (Seeking Health, Bellingham, WA, USA). Bacterial suspensions, at 0.1 optical density (OD) at 600 nm, were inoculated in broth medium and grown in slight motion at 37 °C overnight in aerobic or anaerobic condition, in order to obtain a number of colony‐forming unit (cfu) of ∼ 10^8^/mL, determined by plate counting on medium agar plates. In detail, LGG suspension was inoculated in De Man, Rogosa and Sharpe (MRS) broth and MRS agar medium (Becton Dickinson, Franklin Lakes, NJ, USA) in aerobic condition at 37 °C. *E. coli* ATCC 13762 was cultured in Tryptic Soy (TS) broth and TS agar (OXOID, Basingstoke, Hampshire, UK) in aerobic condition at 37 °C. *B. bifidum* was cultured in anaerobic condition at 37 °C, using MRS broth medium supplemented with 0.25% cysteine/HCl (Sigma‐Aldrich, St. Louis, MO, USA). Bacterial supernatant (SN) of each strain was prepared by centrifugation of the overnight cultures in the specific growth medium at 4000 ***g*** and 4 °C for 10 min and stored in single‐use aliquots at −80 °C until needed.

### 2.3 Flow cytometry

Cells were incubated (30 min at 4 °C) with specific or isotype control Abs. ALOX5, ALOX15A and ALOX15B Abs were from Santa Cruz Biotechnology (Dallas, TX, USA), anti‐GPR32 from Acris (Herford, Germany), anti‐BLT1 from LSBio (Seattle, WA, USA), and anti‐ChemR23 and anti‐FPR1 from R&D Systems (Minneapolis, MI, USA). Cells were analysed with a FACSCalibur cytofluorimeter using the cellquest software (BD Biosciences, Lakes, NJ, USA). When necessary, we performed cell membrane permeabilization using the Cytofix/Cytoperm Kit (BD Biosciences). The receptors followed as indicators of resolution responses were the same modulated by FPR1 in the GC model [10]. The concentration used for flow cytometric staining was that indicated by manufacturers. AnxA1 staining was performed using a primary anti‐AnxA1 goat polyclonal Ab (1 : 500) (R&D Systems) followed by the staining with a secondary anti‐goat Ab Alexa Fluor 488 (Invitrogen, Waltham, MA, USA). The secondary antibody alone was used as a negative matched control.

### 2.4 ELISA and EIA

VEGF‐A contents in culture supernatants were measured in duplicate determinations with a commercially available ELISA (R&D Systems). RvD1, LTB_4_, PGE_2_ and LXB_4_ contents in culture supernatants were measured in triplicate determinations with a commercially available EIA (Cayman Chemical, Ann Arbor, MI, USA) [39]. Cell culture supernatants were assayed, undiluted for autacoid evaluations and diluted 1 : 5 for VEGF‐A release.

### 2.5 Real‐time PCR

Total RNA was isolated and retrotranscribed according to the manufacturer's instructions (Qiagen) as previously described [40]. Real‐time quantitative PCR was performed on iCycler (Bio‐Rad, Hercules, CA, USA) using the PE SYBR Green PCR kit (Applied Biosystems, Foster City, CA, USA) as reported elsewhere [41]. Normalization was performed using *β‐actin* mRNA levels. Primer sequences are listed in Table S1.

### 2.6 Protein studies

Protein extractions and immunoblotting experiments to evaluate signalling pathway activation were performed according to standard procedures [11]. Anti‐phospho‐MAPK, anti‐phospho‐Akt, and anti‐phospho‐STAT3 Abs (1 : 1000) were from Cell Signaling Technology. Antitubulin was from Sigma‐Aldrich (1 : 10 000) (St. Louis, MO, USA), and secondary anti‐mouse and antirabbit Abs coupled to HRP were from Bio‐Rad (1 : 3000).

The expression of angiogenesis‐related proteins in CRC cell culture supernatants was determined using the Human Angiogenesis Array Kit (R&D Systems) according to the manufacturer's instructions. The array allows the detection of 55 angiogenesis‐related proteins by specific capture antibodies spotted onto a nitrocellulose membrane. The data from developed X‐ray film were digitalized and quantified using the ImageJ analysis software [42].

### 2.7 Tubule formation

The formation of network‐like structures by HUVECs (Cell Biologics) on an extracellular matrix (ECM)‐like 3D gel consisting of Matrigel® (BD Biosciences) was performed as previously described and validated [43]. HUVECs (5 × 10^4^) were seeded on a Matrigel matrix in the presence of cell culture supernatants. After incubation, HUVECs underwent differentiation into capillary‐like tube structures. Tubule formation was defined as a structure exhibiting a length four times its width. Network formation was observed using an inverted‐phase contrast microscope (Zeiss, Oberkochen, Germany). Representative fields were taken [43], and the number of branching points counted in five fields was presented as mean ± SD of three experiments.

### 2.8 Wound‐healing assay

For wound‐healing assays, confluent monolayers of HCT116 cells were treated with mitomycin (2 μg·mL^−1^ for 2 h) (Sigma‐Aldrich). The cell monolayers were scraped in three straight lines for each 60‐mm dish with a p10 pipet tip. Cell migration was assessed as previously described [44]. Confluent monolayers were incubated for 30 min with an anti‐GPR32 neutralizing Ab (1 μg·mL^−1^) (GeneTex) or CsH (800 nm) (Sigma‐Aldrich) and then treated with LGG SN or control broth (1 : 30 titration) for 12 h before assessing cell migration.

### 2.9 Statistical analysis

Values from groups were compared using the paired Student *t* test. *P* < 0.05 was considered statistically significant. Clinic–pathologic parameters in relation to *FPR1* or *FPR2* expression were plotted using the cBioPortal database. Coexpression data were obtained according to the cBioPortal online instructions: a log‐rank test was provided to identify the significance of Spearman's correlation coefficient between the mRNA expression z‐scores (RNASeq V2 RSEM) [39].

## 3 Results

### 3.1 FPR1 pharmacologic stimulation sustains a proresolving response in colorectal carcinoma (CRC) cells

We recently described that FPR1 stimulation induces a proresolving program that relies on the expression of enzymes involved in SPM production (*ALOX5*, *ALOX15A* and *ALOX15B*), the release of specific SPMs (RvD1 and LXB_4_) and the expression of SPM receptors (*GPR32*, *ChemR23* and *BLT1*) in gastric cancer (GC) cells [11].

To determine whether FPR1 activates a proresolving program also in colon cancer, we selected two human colorectal carcinoma (CRC) cell lines, HT29 and HCT116 cells. The expression of FPR1 in HCT116 and HT29 cells was assessed by FACS analysis (Fig. S1A). To pharmacologically modulate FPR1, we treated CRC cells with fMLF, an agonist to FPR1 [13], or with cyclosporine H (CsH), an inverse agonist to FPR1 [13], and we analysed the impact of these treatments on SPM pathway.

**Fig. 1:**
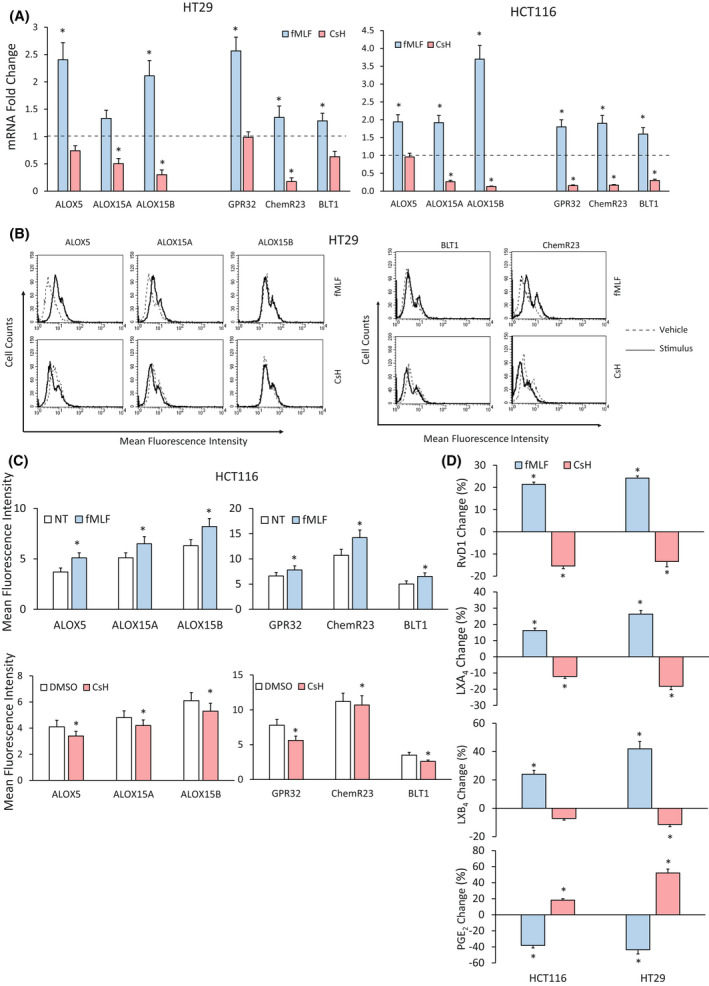
Effects of formyl peptide receptor 1 (FPR1) pharmacologic modulation on specialized proresolving mediator (SPM) biosynthetic machinery of colorectal carcinoma (CRC) cells. (A) *ALOX5*, *ALOX15A*, *ALOX15B*, *GPR32*, *ChemR23* and *BLT1* mRNA fold change in HT29 and HCT116 cells treated with fMLF (10^−9^ m) or CsH (800 nm) for 3 h. Data are represented as mean ± SD of five independent experiments. *P < 0.05 compared with the control (dotted line) by Student's *t* test. (B) ALOX5, ALOX15A, ALOX15B, BLT1 and ChemR23 protein expression levels (mean fluorescence intensity), assessed by cytofluorimetric analysis, in HT29 cells treated with fMLF (10^−9^ m) or CsH (800 nm) for 6 h. A representative experiment of three independent experiments is shown. (C) ALOX5, ALOX15A, ALOX15B, GPR32, ChemR23 and BLT1 protein expression levels (mean fluorescence intensity), assessed by cytofluorimetric analysis, in HCT116 cells treated with fMLF (10^−9^ m) or CsH (800 nm) for 6 h. Data are represented as mean ± SD of three independent experiments. *P < 0.05 compared with the control (NT) by Student's *t* test. (D) Proresolving and proinflammatory autacoid (RvD1, LXA_4_, LXB_4_, PGE_2_) release in HCT116 and HT29 cells treated or not with fMLF (10^−9^ m) or CsH (800 nm) for 12 h. Baseline values of each mediator were in HCT116: RvD1 118 ± 18 pg/10^6^ cells, LXB_4_ 34 ± 5 pg/10^6^ cells, LXA_4_ 485 ± 58 pg/10^6^ cells and PGE_2_ 98 ± 12 pg/10^6^ cells. Baseline values of each mediator were in HT29: RvD1 132 ± 24 pg/10^6^ cells, LXB_4_ 32 ± 4 pg/10^6^ cells, LXA_4_ 462 ± 48 pg/10^6^ cells and PGE_2_ 86 ± 11 pg/10^6^ cells. Data are represented as mean ± SD of changes over baseline levels obtained in five independent experiments. *P < 0.05 compared with the control by Student's *t* test. [Colour figure can be viewed at wileyonlinelibrary.com]

We found that, in HT29 and HCT116 cells, fMLF (10^−9^ m—3‐h treatment) significantly increased, whereas CsH (800 nm—3‐h treatment) significantly decreased, the mRNA expression of enzymes (*ALOX5*, *ALOX15A* and *ALOX15B*) and receptors (*GPR32*, *ChemR23* and *BLT1*) involved in SPM synthesis and recognition (Fig. 1A). In accordance with these observations, the protein levels of SPM enzymes (ALOX5, ALOX15A and ALOX15B) and receptors (GPR32, ChemR23 and BLT1) were significantly induced or reduced, compared with relative controls, in HT29 and HCT116 cells treated for 6 h with fMLF or CsH, respectively, as assessed by cytofluorimetric analysis (Fig. 1B,C).

The activation of SPM synthesis during resolution of inflammation counterbalances the production of proinflammatory lipidic mediators (e.g. prostaglandins and leukotrienes) [8, 39]. In CRC, a crucial proinflammatory and protumorigenic role has been described for PGE_2_ [45]. On the other side, several reports point to an anti‐inflammatory function of LXA_4_ [23, 46]. Thus, we decided to verify whether FPR1 pharmacologic modulation induces changes in PGE_2_ and LXA_4_ release, together with the two SPMs modulated by FPR1 in GC cells (i.e. RvD1 and LXB_4_) [11].

To this purpose, we treated CRC cells with fMLF (10^−9^ m) or with CsH (800 nm) for 12 h and looked for RvD1, LXA_4_, LXB_4_ and PGE_2_ release. We found that fMLF significantly increased, while CsH significantly decreased SPM (RvD1, LXA_4_ and LXB_4_) release in both HT29 and HCT116 cells (Fig. 1D). Consistently, the treatment of HT29 and HCT116 cells with fMLF (10^−9^ m—12 h) significantly reduced, whereas CsH (800 nm—12 h) significantly increased, PGE_2_ release (Fig. 1D).

These data indicate that FPR1 is able to activate in CRC cells an inflammation resolution program, by promoting the induction of SPMs (RvD1, LXA_4_ and LXB_4_), SPM enzymes (ALOX5, ALOX15A and ALOX15B) and SPM receptors (GPR32, ChemR23 and BLT1).

### 3.2 FPR1 pharmacologic modulation controls the angiogenic potential of CRC cells

We previously found that FPR1 ablation/pharmacological inhibition caused a drop in the endogenous levels of SPMs and a concomitant increase in the angiogenic potential of GC cells. We also found that SPMs control the production of angiogenic mediators in GC cells, since the exogenous administration of SPMs (RvD1 or LXB_4_) to FPR1‐depleted GC cells could suppress their increased angiogenic potential [11].

**Fig. 2:**
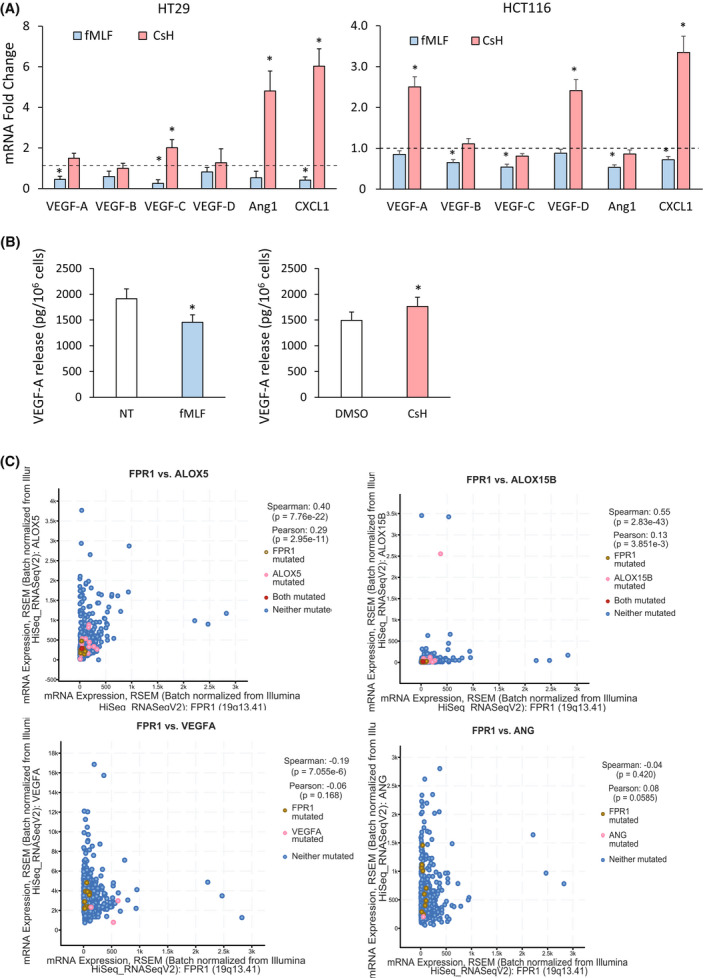
Effects of formyl peptide receptor 1 (FPR1) pharmacologic modulation in colorectal carcinoma (CRC) cells on angiogenic response. (A) *VEGF‐A*, *VEGF‐B*, *VEGF‐C*, *VEGF‐D*, *Ang1* and *CXCL1* mRNA fold change in HT29 and HCT116 cells treated with fMLF (10^−9^ m) or CsH (800 nm) for 3 h. Data are represented as mean ± SD of five independent experiments. *P < 0.05 compared with the control (dotted line) by Student's *t* test. (B) VEGF‐A release in HCT116 cells treated with fMLF (10^−9^ m) or CsH (800 nm) or the relative controls for 12 h. Data are represented as mean ± SD of five independent experiments. *P < 0.05 compared with the control by Student's *t* test. (C) Correlation between the mRNA expression levels of the indicated markers in 594 patients affected by colorectal adenocarcinoma. Spearman's factor, Pearson's factor and the relative p are indicated. [Colour figure can be viewed at wileyonlinelibrary.com]

To investigate whether FPR1 acts as a negative modulator of angiogenesis also in CRC cells, we treated HT29 and HCT116 cells with fMLF (10^−9^ m) or with CsH (800 nm) for 3 h and measured the mRNA expression levels of several proangiogenic mediators (*VEGF‐A*, *VEGF‐B*, *VEGF‐C*, *VEGF‐D*, *Ang1* and *CXCL1*). We observed that fMLF induced a reduction of mRNA levels for *VEGF‐A*, *VEGF‐C* and *CXCL1* in HT29 cells and for *VEGF‐B*, *VEGF‐C*, *Ang1* and *CXCL1* in HCT116 cells (Fig. 2A). Consistently, CsH treatment significantly increased mRNA expression of proangiogenic molecules in both HT29 and HCT116 cells (Fig. 2A). Moreover, the release of VEGF‐A was significantly lower in HCT116 treated for 12 h with fMLF (10^−9^ m) and significantly higher in the same cells treated with CsH (800 nm) compared to relative controls (Fig. 2B). Similar results were obtained in HT29 cells (not shown).

To corroborate our results in CRC cultures, we verified the mRNA coexpression data present in the publicly available cBioPortal for Cancer Genomics database (http://www.cbioportal.org) [47, 48]. Data on 594 colorectal adenocarcinoma revealed that *FPR1* mRNA levels significantly and directly correlated with mRNA expression levels of the proresolving factors *ALOX5* and *ALOX15B* (Fig. 2C). Consistently, *FPR1* mRNA levels inversely correlated with mRNA levels of two key angiogenic mediators [*VEGF‐A* and *angiopoietin 1* (*Ang*)] (Fig. 2C).

Finally, in order to search for information on the clinic–pathologic role of FPRs and/or proresolving pathways in CRC, we queried the cBioPortal for Cancer Genomics database (http://www.cbioportal.org): mRNA levels of *FPR1* showed a statistic trend of association with the Overall Survival Status (*P* = 0.11) and a direct and statistically significant association with the disease‐free months (Fig. S2). No statistically significant association with the two parameters was found for *FPR2*, suggesting that FPR1 in CRC plays a nonredundant role similar to that observed in GC [11].

These data support the hypothesis that FPR1 is responsible for a proresolving and antiangiogenic response in CRC cells.

### 3.3 FPR1 genetic ablation reduces the proresolving activities and increases the angiogenic potential of CRC cells

To confirm the results presented in 3.1 and 3.2 paragraphs, we generated HCT116 cells stably transfected either with *FPR1*‐targeting short hairpin RNAs (HCT116 shFPR1) or with nontargeting short hairpin RNAs (HCT116 shCTR). We identified various clones expressing low levels of the receptor (Fig. S1B).

**Fig. 3:**
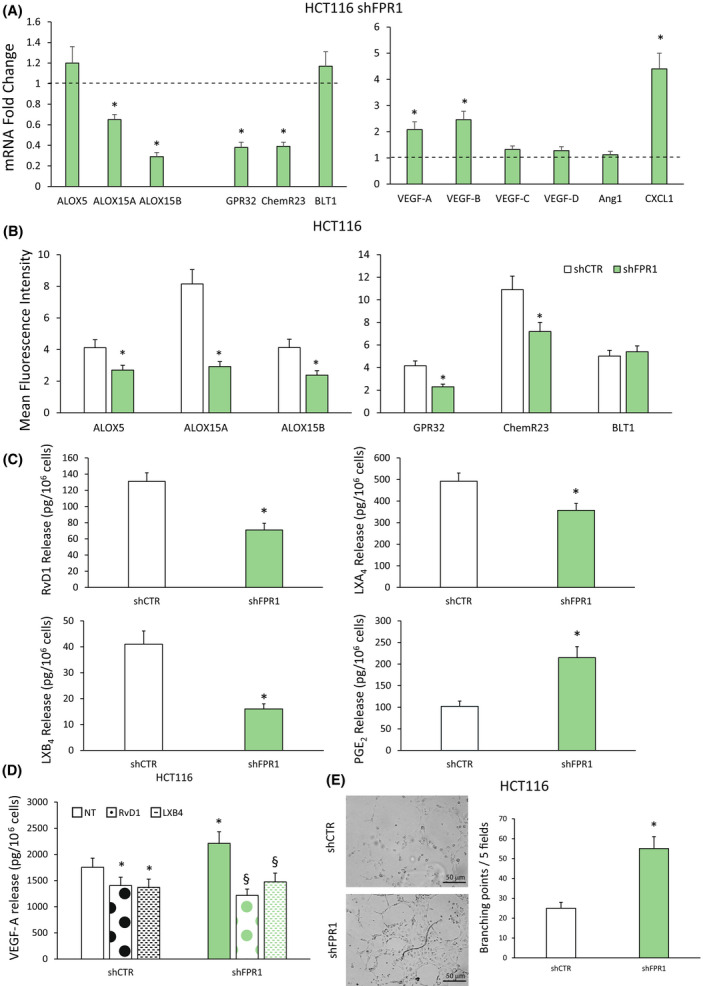
Effects of *formyl peptide receptor 1* (*FPR1*) silencing on specialized proresolving mediator (SPM) biosynthetic machinery and angiogenic potential of colorectal carcinoma (CRC) cells. (A) *ALOX5*, *ALOX15A*, *ALOX15B*, *GPR32*, *ChemR23*, *BLT1*, *VEGF‐A*, *VEGF‐B*, *VEGF‐C*, *VEGF‐D*, *Ang1* and *CXCL1* mRNA fold change in HCT116 cells silenced for *FPR1* (HCT116 shFPR1, three clones) compared to cells transfected with nontargeting short hairpin RNAs (shCTR cells—dotted line, a mass population). Data are represented as mean ± SD of five independent experiments. *P < 0.05 compared with the control (dotted line) by Student's *t* test. (B) ALOX5, ALOX15A, ALOX15B, GPR32, ChemR23 and BLT1 expression levels (mean fluorescence intensity), assessed by cytofluorimetric analysis, in HCT116 shFPR1 (three clones) cells and the relative control (shCTR, a mass population). Data are represented as mean ± SD of five independent experiments. *P < 0.05 compared with the relative control by Student's *t* test. (C) Proresolving and proinflammatory autacoid (RvD1, LXA_4_, LXB_4_, PGE_2_) release in HCT116 shCTR (a mass population) or shFPR1 (three clones). Data are represented as mean ± SD of five independent experiments. *P < 0.05 compared with the relative control by Student's *t* test. (D) VEGF‐A release in HCT116 shCTR cells (a mass population) or shFPR1 cells (three clones) treated or not (NT) with RvD1 (1 nm) or LXB_4_ (1 nm) for 12 h. Data are represented as mean ± SD of five independent experiments. *P < 0.05 compared with shCTR NT by Student's *t* test. ^§^ *P* < 0.05 compared with the relative control by Student's *t* test. (E) Human umbilical vein endothelial cells (HUVECs) were cultured in the presence of cell culture conditioned media (CM) from HCT116 shCTR (a mass population) or shFPR1 (three clones) (10× magnifications) in a 24‐well plate. After 12 h, cells were fixed with ice‐cold 70% ethanol, and tubule formation was evaluated. Sample images and a quantification of the angiogenic response are reported. Scale bar 50 μm. Data are represented as mean ± SD of three independent experiments. *P < 0.05 compared with the control by Student's *t* test. [Colour figure can be viewed at wileyonlinelibrary.com]

By real‐time PCR, we found that genetic ablation of *FPR1* significantly reduced mRNA level of proresolving enzymes (*ALOX15A* and *ALOX15B*) and receptors (*GPR32*, *ChemR23*) compared with control cells (Fig. 3A). Moreover, HCT116 shFPR1 cells exhibited an increase of mRNA levels for angiogenic mediators compared with controls (Fig. 3A).

The genetic ablation of *FPR1* significantly decreased the protein levels of ALOX5, ALOX15A, ALOX15B, GPR32 and ChemR23, but not BLT1 compared with HCT116 shCTR cells, as assessed by cytofluorimetric analysis (Fig. 3B). Moreover, the amount of RvD1, LXB_4_ and LXA_4_ released was significantly reduced in HCT116 shFPR1 compared with that in shCTR cells (Fig. 3C). Furthermore, *FPR1* silencing in HCT116 cells caused the release of higher levels of PGE_2_ compared with that of controls (Fig. 3C). In accordance, these cells displayed increased constitutive release of VEGF‐A (Fig. 3D).

The formation of capillary‐like tube structures in the extracellular matrix by endothelial cells (ECs) is a classic method to measure angiogenesis *in vitro* [49]. To investigate whether differences in FPR1 expression/activation control functional angiogenic properties of CRC cells, we studied the ability of CRC cell conditioned media (CM) to induce human umbilical vein endothelial cell (HUVEC) network formation on a Matrigel substrate. In particular, we evaluated tubule formation *in vitro* in response to CM from HCT116 cells silenced or not for *FPR1* (shCTR vs shFPR1). As shown in Fig. 3E, HUVECs plated in the presence of CM from HCT116 shCTR cells formed only a few tube structures at 12 h. On the contrary, when the endothelial cells were plated in the presence of CM from HCT116 shFPR1, a significantly higher number of formed tube structures were observed compared with that induced by shCTR CM (Fig. 3E).

To assess whether the increased proangiogenic potential of *FPR1*‐silenced HCT116 cells could be due to the defective SPM biosynthesis of these cells, we added back LXA_4_ (1 nm), RvD1 (1 nm) or LXB_4_ (1 nm) to HCT116 shFPR1 and shCTR cells for 3 h and evaluated their proangiogenic activity. By means of real‐time PCR, we observed that LXA_4_ had no effects on proangiogenic mediator expression (Fig. S3). Thus, although it has been described that LXA_4_ exerts a strong anti‐inflammatory potential in CRC [23, 46], our results demonstrate that it has no effect on the modulation of CRC cell angiogenic potential.

RvD1 and LXB_4_ were able to reduce the expression of proangiogenic mRNAs, in HCT116 shFPR1 and, to a lesser extent, in HCT116 shCTR cells (Fig. S4A). Consistently, as shown in Fig. 3D, we found that RvD1 and LXB_4_ significantly reduced VEGF‐A protein release in both HCT116 shFPR1 and, to a lesser extent, HCT116 shCTR cells.

Finally, RvD1 and LXB_4_ displayed the ability to restore the expression of ALOXs and SPM receptor mRNAs in HCT116 shFPR1 cells (Fig. S4B). The RvD1‐ and LXB_4_‐induced upregulation of SPM enzymes and receptors was also confirmed at the protein level by FACS analysis (Fig. S4C,D).

These results demonstrate that FPR1 exerts an antiangiogenic effect in CRC cells through the modulation of SPM production.

### 3.4 *Lactobacillus rhamnosus* GG (LGG) supernatant sustains proresolving and antiangiogenic responses in CRC cells

Previous studies showed that *Lactobacillus rhamnosus* GG (LGG), a colonic commensal bacterium and one of the most used probiotic strains [30], exerts a homeostatic function in intestinal mucosa [31, 32, 33], decreases levels of procarcinogenic metabolites, reduces chronic inflammation associated with intestinal neoplastic transformation and inhibits the proliferation of malignant cells [30, 35, 36]. Some of these LGG functions were associated with its ability to interact with FPR1 [32]. Thus, we hypothesized that LGG protective effect in normal intestinal mucosa and LGG anticancer activities in CRC cells could be linked to a proresolving and antiangiogenic response activated by the bacteria.

In order to verify our hypothesis, we treated HT29 and HCT116 cells with LGG supernatant (SN) (1 : 30 titration) for 3 h, or with the same dilution of the culture broth, and evaluated, by real‐time PCR, the expression levels of proresolving pathways' components and of proangiogenic markers. The 1 : 30 titration was chosen as optimal one after testing different dilutions (1 : 10–1 : 100) of LGG SN in cell culture media in order to balance pH changes induced by LGG SN and its functional activity (not shown). We found that LGG SN induced, in both CRC cells, a significant increase of *ALOX15A*, *ALOX15B*, *GPR32*, *ChemR23* and *BLT1* mRNA levels (Fig. S5A) and a statistically significant decrease of proangiogenic mediator mRNA levels (*VEGF‐C*, *VEGF‐D*, *Ang1* and *CXCL1*) (Fig. S5B) when compared to control (culture broth).

**Fig. 4:**
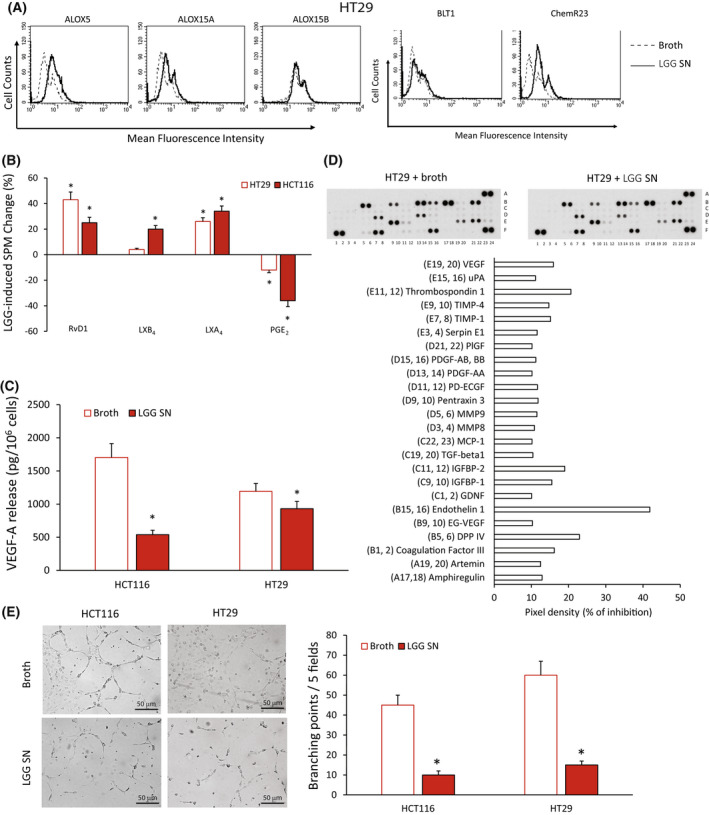
Effects of *Lactobacillus rhamnosus* GG (LGG) supernatant (SN) on specialized proresolving mediator (SPM) biosynthetic machinery and angiogenic potential of colorectal carcinoma (CRC) cells. (A) ALOX5, ALOX15A, ALOX15B, BLT1, and ChemR23 protein expression levels (mean fluorescence intensity), assessed by cytofluorimetric analysis, in HT29 cells treated with *Lactobacillus rhamnosus* GG (LGG) supernatant (SN) or its control broth—1 : 30 titration for 3 h. A representative experiment of three independent experiments is shown. (B) Proresolving and proinflammatory autacoid (RvD1, LXB_4_, LXA_4_, PGE_2_) release in HT29 and HCT116 cells treated with LGG SN—1 : 30 titration for 12 h over control (broth). Baseline values of each mediator were in HCT116 cells: RvD1 122 ± 15 pg/10^6^ cells, LXB_4_ 38 ± 4.2 pg/10^6^ cells, LXA_4_ 501 ± 54 pg/10^6^ cells and PGE_2_ 101 ± 11 pg/10^6^ cells. Baseline values of each mediator were in HT29 cells: RvD1 128 ± 27 pg/10^6^ cells, LXB_4_ 36 ± 5 pg/10^6^ cells, LXA_4_ 475 ± 52 pg/10^6^ cells and PGE_2_ 82 ± 9.8 pg/10^6^ cells. Data are represented as mean ± SD of changes over baseline levels obtained in five independent experiments. *P < 0.05 compared with the control by Student's *t* test. (C) VEGF‐A release in HCT116 and HT29 cells treated with LGG SN—1 : 30 titration or the control culture broth for 12 h. Data are represented as mean ± SD of five independent experiments. *P < 0.05 compared with the control by Student's *t* test. (D) Analysis of proteins in conditioned media (CM) from HT29 cells treated with LGG SN or its control broth (1 : 30 titration) using angiogenesis‐associated protein antibody arrays. The mean of protein pixel density for each angiogenesis‐related protein, normalized for the reference spots, was calculated and compared with the relative control. The array images and the relative quantitative profiles of protein levels are shown. (E) Human umbilical vein endothelial cells (HUVECs) were cultured in the presence of cell culture conditioned media (CM) from HCT116 or HT29 cells treated with LGG SN or the control culture broth (1 : 30 titration) (10× magnification) in a 24‐well plate. After 12 h, cells were fixed with ice‐cold 70% ethanol, and tubule formation was evaluated. Sample images and a quantification of the angiogenic response are reported. Scale bar 50 μm. Data are represented as mean ± SD of three independent experiments. *P < 0.05 compared with the control (broth) by Student's *t* test. [Colour figure can be viewed at wileyonlinelibrary.com]

We confirmed these observations at the protein level: the treatment of HT29 cells with LGG SN for 6 h significantly increased ALOX5, ALOX15A, ALOX15B, BLT1 and ChemR23 protein levels (Fig. 4A). Similar results were obtained in HCT116 cells (not shown). Consistently with the induction of enzymes, LGG SN (12 h treatment) induced a significant increase in SPM release (RvD1, LXB_4_, LXA_4_) and a significant decrease in PGE_2_ levels in both HT29 and HCT116 cells (Fig. 4B).

To confirm the antiangiogenic effect of LGG in CRC cells, we evaluated, by an ELISA assay, VEGF‐A release in HCT116 and HT29 cells treated for 12 h with LGG SN or broth as a control. We found that LGG SN was able to significantly reduce VEGF‐A release in both CRC cells compared with the control broth (Fig. 4C). Furthermore, we evaluated the expression of several other angiogenic proteins using dedicated antibody arrays incubated with CM from HT29 cells treated for 12 h with LGG SN or the relative control (broth) [39]. LGG SN (1 : 30 titration) treatment in HT29 cells downregulated, with changes superior to 10%, the levels of amphiregulin, artemin, coagulation factor III, DPP IV, EG‐VEGF, endothelin 1, GDNF, IGFBP‐1, IGFBP‐2, TGF‐beta1, MCP‐1, MMP8, MMP9, pentraxin 3, PD‐ECGF, PDGF‐AA, PDGF‐AB and BB, PlGF, Serpin E1, TIMP‐1, TIMP‐4, Thrombospondin 1, uPA and VEGF (Fig. 4D).

We then evaluated the ability of LGG SN to modulate CRC cell functional angiogenic potential. HUVECs plated on wells coated with Matrigel with the addition of CM from HCT116 or HT29 cells treated with control culture broth (1 : 30 titration) formed a characteristic capillary‐like network at 12 h. On the contrary, when the cells were plated on Matrigel with the addition of CM from HCT116 or HT29 treated with LGG SN (1 : 30 titration), a significantly lower number of tube structures were observed (Fig. 4E).

Finally, to verify whether the antiangiogenic potential of LGG SN depends on SPM activity, we used a GPR32 neutralizing antibody (Ab) that inhibits the activity of its ligand RvD1. For this purpose, HCT116 cells were stimulated with LGG SN in the presence or absence of the anti‐GPR32 (1 μg·mL^−1^) Ab, and their angiogenic potential was evaluated. As shown in Fig. S6, at the concentration used for this experiment, the neutralizing anti‐GPR32 Ab was able to significantly reduce the LGG‐mediated inhibition of the angiogenic potential of CRC cells. The anti‐GPR32 effect was partial, as expected, due to its ability to block only RvD1 effects and not that of other SPMs (Fig. S6).

These data suggest that LGG is able to induce a proresolving response and a following antiangiogenic effect in CRC cells.

### 3.5 The proresolving and antiangiogenic properties of LGG are not common to other commensal bacteria

In order to verify the specificity of action of LGG, we asked whether *Escherichia coli* (*E. coli*), as example of a commensal nonprobiotic strain [50], or *Bifidobacterium bifidum* (*B. bifidum*), as other lactic acid probiotic strain [51], could sustain a proresolving and antiangiogenic response in CRC cells.

**Fig. 5:**
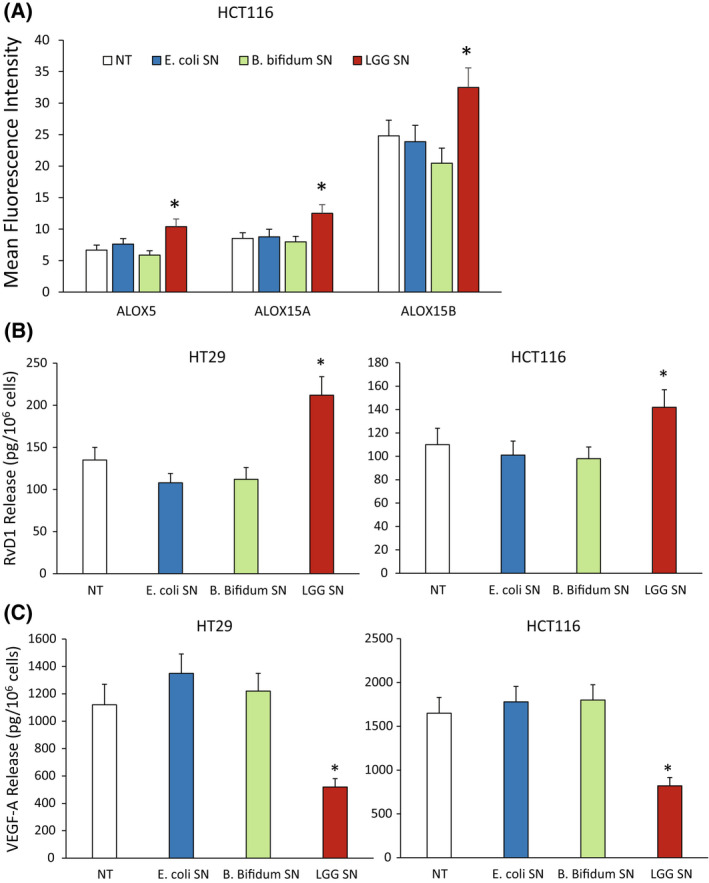
Effects of other commensal bacterial strain on specialized proresolving mediator (SPM) biosynthetic machinery and angiogenic potential in colorectal carcinoma (CRC) cells. (A) ALOX5, ALOX15A and ALOX15B protein expression levels (mean fluorescence intensity), assessed by cytofluorimetric analysis, in HCT116 cells treated or not (NT) with *Escherichia coli* (*E. coli*), *Bifidobacterium bifidum* (*B. bifidum*) and *Lactobacillus rhamnosus* GG (LGG) supernatant (SN) (1 : 30 titration) for 6 h. Data are represented as mean ± SD of three independent experiments. *P < 0.05 compared with the relative control by Student's *t* test. (B) RvD1 release from HT29 and HCT116 cells treated or not (NT) for 12 h with *E. coli*, *B. bifidum* and LGG SN (1 : 30 titration). Data are represented as mean ± SD of three independent experiments. *P < 0.05 compared with the control by Student's *t* test. (C) VEGF‐A release from HT29 and HCT116 cells treated or not (NT) for 12 h with *E. coli*, *B. bifidum* and LGG SN (1 : 30 titration). Data are represented as mean ± SD of three independent experiments. *P < 0.05 compared with the control by Student's *t* test. [Colour figure can be viewed at wileyonlinelibrary.com]

To this aim, we treated HT29 and HCT116 cells with E. coli or B. bifidum SN (1 : 30 titration) and verified their effects on ALOX expression, RvD1 and VEGF‐A release. By flow cytometric analysis, we observed that neither *E. coli* nor *B. bifidum* SN were able to increase the level of ALOX5, ALOX15A and ALOX15B proteins in HCT116 cells, while LGG did it (Fig. 5A). Similar results were obtained also in HT29 cells (not shown). Consistently, LGG SN significantly induced RvD1 release, while *E. coli* and *B. bifidum* SN did not (Fig. 5B) both in HT29 and in HCT116 CRC cells. Finally, we verified the effects of *E. coli* or *B. bifidum* SN on VEGF‐A release. Neither *E. coli* nor *B. bifidum* SN were able to reduce VEGF‐A release from HT29 and HCT116 cells (Fig. 5C).

Altogether, these data support the evidence that the activation of a proresolving and antiangiogenic response in CRC cells is not general and common to all the commensal or to all the lactic acid bacteria.

### 3.6 *Lactobacillus rhamnosus* GG (LGG)‐mediated proresolving response requires FPR1

It has been reported that LGG depends on the expression of *FPR1* for its activity in colon cells [32, 52]. To provide evidence that LGG‐mediated proresolving and antiangiogenic responses in CRC cells require FPR1, we treated HCT116 shFPR1 and shCTR with LGG SN or with the control broth and we evaluated the production of proresolving and proangiogenic factors.

**Fig. 6:**
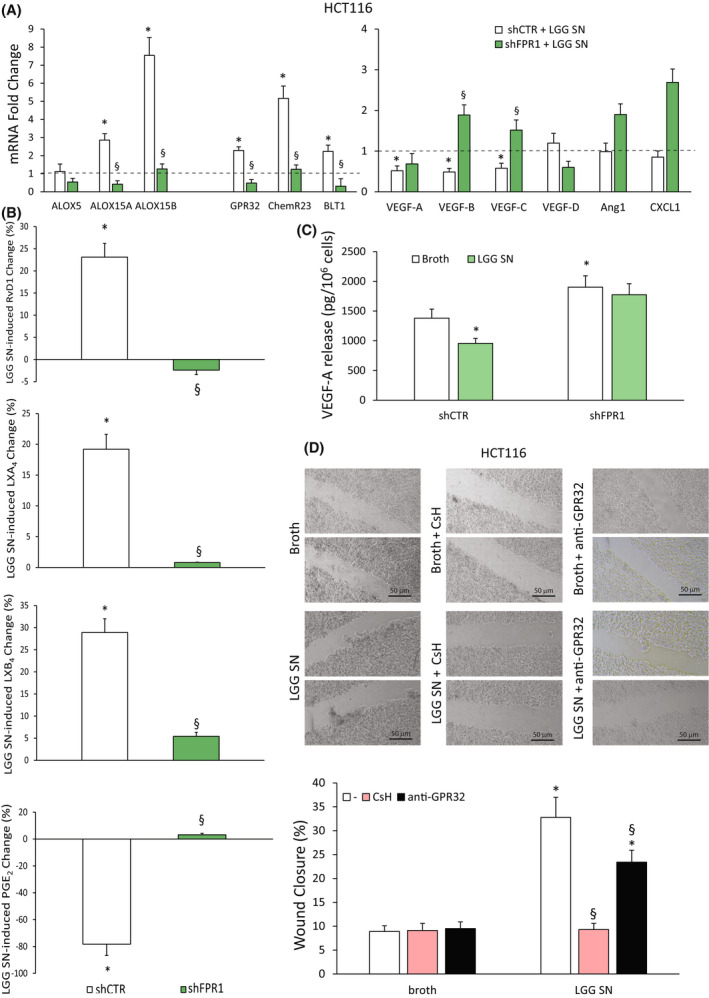
Dependence of *Lactobacillus rhamnosus* GG (LGG) supernatant (SN) effects on formyl peptide receptor 1 (FPR1) expression in colorectal carcinoma (CRC) cells. (A) *ALOX5*, *ALOX15A*, *ALOX15B*, *GPR32*, *ChemR23*, *BLT1*, *VEGF‐A*, *VEGF‐B*, *VEGF‐C*, *VEGF‐D*, *Ang1* and *CXCL1* mRNA fold change in HCT116 cells silenced for FPR1 (HCT116 shFPR1, three clones) or in control cells transfected with nontargeting short hairpin RNAs (shCTR cells, a mass population) upon treatment for 3 h with *Lactobacillus rhamnosus* GG (LGG) supernatant (SN)—1 : 30 titration. Data are represented as mean ± SD of five independent experiments. *P < 0.05 compared with the control (broth—dotted line) by Student's *t* test, ^§^ *P* < 0.05 compared with the relative control by Student's *t* test. (B) Proresolving and proinflammatory autacoid (RvD1, LXA_4_, LXB_4_, PGE_2_) release over control in HCT116 shCTR (a mass population) or shFPR1 (three clones) upon treatment for 12 h with LGG SN—1 : 30 titration. Baseline values of each mediator were in HCT116 shCTR cells: RvD1 128 ± 18 pg/10^6^ cells, LXB_4_ 41 ± 5 pg/10^6^ cells, LXA_4_ 492 ± 51 pg/10^6^ cells and PGE_2_ 98 ± 13 pg/10^6^ cells. Baseline values of each mediator were in HCT116 shFPR1 cells: RvD1 68 ± 7 pg/10^6^ cells, LXB_4_ 18 ± 3 pg/10^6^ cells, LXA_4_ 346 ± 42 pg/10^6^ cells and PGE_2_ 214 ± 28 pg/10^6^ cells. Data are represented as mean ± SD of changes over baseline levels obtained in five independent experiments. *P < 0.05 compared with the broth control by Student's *t* test, ^§^ *P* < 0.05 compared with the relative control by Student's *t* test. (C) VEGF‐A release in HCT116 shCTR (a mass population) or shFPR1 (three clones) cells treated with LGG SN—1 : 30 titration or the culture broth for 12 h. Data are represented as mean ± SD of five independent experiments. *P < 0.05 compared with shCTR broth by Student's *t* test. (D) Wound‐healing assay of HCT116 cells in the presence of LGG SN (1 : 30 titration) or the same dilution of culture broth for 12 h. Cells were pretreated or not for 30 min with CsH (800 nm) or a neutralizing anti‐GPR32 antibody (1 μg·mL^−1^). Representative photograms and a quantitative evaluation of the wound closure are shown. Scale bar 50 μm. Values represent the average of triplicate experiments ± SD. *P < 0.05 compared with broth alone by Student's *t* test. ^§^ *P* < 0.05 compared with the relative control by Student's *t* test. [Colour figure can be viewed at wileyonlinelibrary.com]

LGG SN (1 : 30 titration—3 h) promoted a significant increase of *ALOX5*, *ALOX15A* and *ALOX15B* mRNA levels in shCTR but not in shFPR1 CRC cells (Fig. 6A). Similar results were obtained for the receptors *GPR32*, *ChemR23* and *BLT1* (Fig. 6A). Consistently, LGG SN induces the reduction of pro‐angiogenic mediators (*VEGF‐A, ‐B, ‐C*) only in shCTR but not in shFPR1 CRC cells (Fig. 6A).

To determine whether LGG depends on FPR1 also for its induction of SPM release, we treated shCTR and shFPR1 HCT116 cells with LGG SN for 12 h. We found that HCT116 shFPR1 treated with LGG SN did not display the increase in RvD1, LXA_4_ and LXB_4_ release observed in control cells (Fig. 6B). As expected, LGG SN caused a significant reduction in PGE_2_ release in shCTR but not in shFPR1 HCT116 cells (Fig. 6B).

To confirm that also the antiangiogenic effect of LGG in CRC cells depends on *FPR1*, we evaluated the VEGF‐A release in HCT116 shFPR1 and shCTR cells in the presence or absence of LGG SN after 12 h. As shown in Fig. 6C, VEGF‐A release was higher in HCT116 shFPR1 than in controls; LGG SN treatment was able to significantly reduce VEGF‐A release in shCTR but not in shFPR1 HCT116 cells (Fig. 6C).

Taken together, these data demonstrate that the proresolving and antiangiogenic activities of LGG require FPR1.

It has been described that one of the most important homeostatic functions of LGG is mediated by its ability to sustain restitution of injured intestinal epithelial monolayers by interacting with FPR1 [31, 52]. Here, we verified whether the ability of LGG to induce CRC epithelial cell restitution through the activation of FPR1 is dependent, at least in part, on the activation of proresolving pathways. To this aim, we performed a wound‐healing assay on HCT116 in the presence or absence of LGG SN, CsH (800 nm) or a neutralizing anti‐GPR32 Ab (1 μg·mL^−1^). As shown in Fig. 6D, LGG SN elicits a significant movement of HCT116 cells following injury, which was completely abolished by CsH, confirming the dependence of LGG effects on FPR1 (Fig. 6D). Interestingly, the neutralizing antibody against GPR2 was able to significantly reduce (about 30%) the LGG SN‐induced migration, suggesting that the effects of LGG SN on FPR1 in terms of wound closure imply, at least in part, the production of proresolving mediators (i.e. RvD1).

In other experimental models, the activation of a proresolving response goes through the induction, not only of lipidic SPMs but also of proresolving mediators of a different type (e.g. AnxA1) [53]. In order to verify the possibility that LGG and FPR1 could induce AnxA1 expression in CRC cells, we treated both HT29 and HCT116 cells with LGG SN (1 : 30 titration), fMLF (10^−9^ m) or the three SPMs [RvD1 (1 nm), LXB_4_ (1 nm) and LXA_4_ (1 nm)] for 12 h and verified the expression levels of AnxA1, a potent endogenous proresolving and immunomodulatory protein [5].

FACS analysis shows that both fMLF and LGG SN induced an increase in the protein expression of AnxA1 in CRC cells (Fig. S7). Similarly, the treatment with RvD1, LXB_4_ and LXA_4_ increased AnxA1 protein expression levels in CRC cells (Fig. S7). These observations suggest that FPR1 activation sustains a proresolving response that includes AnxA1 and that SPMs could further stimulate *AnxA1* expression in a feed‐forward loop [53].

These results confirm previous observations indicating that LGG mediates a homeostatic function in colonic mucosa requiring *FPR1* [31, 32]. Moreover, our data indicate that this FPR1 function is maintained also in CRC cells, and is dependent on its ability to sustain a proresolving response.

### 3.7 FPR1 activation of proresolving program requires MAPK activation

We then explored the signalling pathways, which are involved in the proresolving response of CRC cells to FPR1 activation mediated by LGG or fMLF.

To this aim, we treated HCT116 cells with fMLF (10^−9^ m) or LGG SN (1 : 30 titration) and the relative controls (not‐treated or broth alone, respectively) and harvested at different time points. fMLF has been demonstrated to classically activate the ERK, PI3K/Akt and STAT3 pathways [54, 55]; thus, we verified the expression levels of phospho‐MAPK, phospho‐Akt, and phospho‐STAT3 in order to evaluate the activated forms of these proteins.

**Fig. 7:**
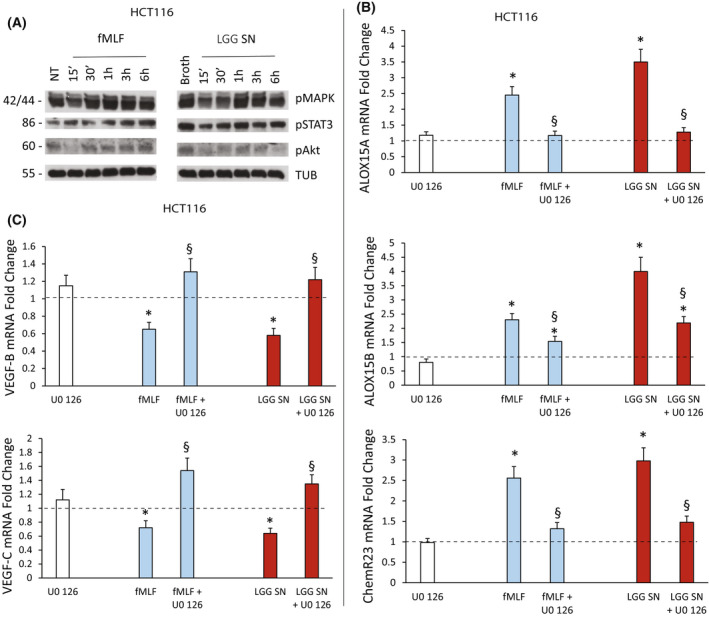
Signaling pathway activation upon fMLF or *Lactobacillus rhamnosus* GG (LGG) supernatant (SN) treatment of colorectal carcinoma (CRC) cells. (A) Activation of downstream signaling pathways in HCT116 cells induced by fMLF (10^−9^ m) or *Lactobacillus rhamnosus* GG (LGG) supernatant (SN) (1 : 30 titration) or the relative controls [not‐treated (NT) or broth alone, respectively]. Total cell lysates were prepared at various time points after stimulation in serum‐free medium. Immunoblots were probed with the indicated phosphospecific Abs. Antitubulin was used for normalization. The figure shows the results of a representative experiment from among three different preparations. (B) *ALOX15A*, *ALOX15B* and *ChemR23* mRNA fold change in HCT116 cells treated with fMLF (10^−9^ m) or LGG SN (1 : 30 titration) for 3 h following or not cell preincubation with U0 126 (10 μm) for 30 min. Data are represented as mean ± SD of five independent experiments. *P < 0.05 compared with the negative control (dotted line) by Student's *t* test; ^§^ *P* < 0.05 compared with the relative control treatment by Student's *t* test. (C) *VEGF‐B* and *VEGF‐C* mRNA fold change in HCT116 cells treated with fMLF (10^−9^ m) or LGG SN (1 : 30 titration) for 3 h following or not cell preincubation with U0 126 (10 μm) for 30 min. Data are represented as mean ± SD of five independent experiments. *P < 0.05 compared with the negative control (dotted line) by Student's *t* test; ^§^ *P* < 0.05 compared with the relative control treatment by Student's *t* test. [Colour figure can be viewed at wileyonlinelibrary.com]

As shown in Fig. 7A, in HCT116 cells a significant activation of MAPK and STAT3 was observed in response to fMLF, while no significant activation levels of Akt were detected. LGG SN, when compared to the broth alone at the same titration (1 : 30), activated MAPK but not STAT3 signalling (Fig. 7A). Thus, the common proresolving and antiangiogenic properties of fMLF and LGG SN could be due to MAPK activation.

To verify this hypothesis, we treated HCT116 cells with fMLF (10^−9^ m) or LGG SN (1 : 30 titration) and the relative controls (not‐treated or broth alone, respectively) in the presence or absence of U0 126, a selective inhibitor of mitogen‐activated protein kinase kinase [56]. Figure 7B shows that fMLF and LGG SN significantly upregulated *ALOX15A*, *ALOX15B* and *ChemR23* expression levels and that the preincubation of cells with U0 126 partially or completely reverted these effects. Consistently, *VEGF‐B* and *VEGF‐C* were significantly reduced by fMLF and LGG SN treatments and these effects were not detectable in cells pretreated with U0 126 (Fig. 7C).

These experiments demonstrated that the proresolving and antiangiogenic responses of fMLF and LGG require MAPK signalling activation.

## 4 Discussion

The homeostasis of intestinal mucosa is tightly regulated by mechanisms able to perceive bacterial species distinguishing between pathogenic and commensal ones, triggering an inflammatory antibacterial and a tolerogenic proresolving response, respectively [1, 57]. Formyl peptide receptors (FPRs), a family of pattern recognition receptors, can recognize several bacterial products and trigger either inflammation or its resolution [13, 14], being optimal candidates to the role of central regulators of intestinal mucosa homeostasis [14, 32, 58].

It has been reported that intestinal epithelial cells sense commensal bacteria using various receptors, including FPRs; as a consequence of this, an increase in barrier function, and improved resolution of epithelial wounds are observed [31, 52, 58, 59]. For instance, it has been demonstrated that the commensal bacteria *Lactobacillus rhamnosus* GG (LGG), by activating FPR1, influence intestinal epithelial homeostatic signalling and sustain epithelial cell motility enhancing wound restitution [31, 52]. Furthermore, several studies point to an important role of intestinal microbiota, not only in the physiology of intestinal mucosa but also in eliciting a protective antitumour response by both acting directly on cancer cells and modulating the immune response to them [26, 28, 30, 60].

We recently showed that the genetic ablation of *FPR1* caused an increase in proinflammatory, angiogenic and tumorigenic potential in gastric cancer (GC) cells [10, 11]. We further showed that these functions of FPR1 are mediated by its ability to sustain the expression and function of proresolving pathways [11]. We also demonstrated that Toll‐like receptor 7 (TLR7) displays similar proresolving and antiangiogenic properties in non‐small‐cell lung cancer cells [39]. Here, we demonstrated, for the first time, that *FPR1* exerts a similar function also in the intestinal mucosa. Taken together, our results sustain the hypothesis that specific PRRs could exert homeostatic and proresolving functions in different tissues, which need to balance injuries and inflammatory insults.

More in detail, we showed that FPR1 activation mediated by formyl peptides, which are the main natural ligands to FPR1 [13], or by LGG SN, induces the expression of enzymes and receptors involved in proresolving responses, and the release of a significantly higher amount of SPMs (RvD1, LXB_4_, LXA_4_) at the expense of proinflammatory lipid mediators (PGE_2_). Although we focused our attention specifically on lipidic proresolving autacoids, the proresolving response in CRC cells mediated by FPR1 could be more general, since we also verified that fMLF and LGG are able to induce the expression in CRC cells of *AnxA1*, a proteic proresolving mediator [53].

The physiology of this type of response is of paramount importance, if one takes into account that SPMs act *in vitro* at an optimal concentration in the low nanomolar range. Indeed, we observed that the antiangiogenic properties of SPMs are detectable at 0.5–1 nm concentration. By means of EIA, we detected basal concentration of RvD1 released by 10^6^ of two distinct CRC cells around 300 pm; LXB_4_ was constitutionally released at ∼ 80 pm, while LXA_4_ was around 1.5 nm. The stimulation of CRC cells with LGG increased RvD1 release of around 40%, LXB_4_ of 20% and LXA_4_ of 30%, thus allowing to reach in cell culture media, and presumably in the gut, optimal and active concentrations of each SPM. Furthermore, it should be considered that while we identified 1 nm as the optimal concentration of each SPM alone to inhibit angiogenesis, in the gut the interaction of LGG product(s) with intestinal epithelial cells allows the contemporary production of several SPMs, which could act in synergy.

Although we have still not identified the bacterial products secreted in the LGG SN and responsible for FPR1 stimulation, it is likely that LGG‐derived formylated peptides are the mediators of the observed effects. However, we cannot exclude the presence in LGG SN of other FPR1 agonist(s). Whatever the case, the activity of secreted FPR1 ligands in LGG SN suggests that, in the physiology of the gut, LGG could exert its homeostatic/protective effects on intestinal mucosa by releasing several factors in the extracellular space.

Consistently with previous observations demonstrating that LGG sustains intestinal epithelial restitution via an FPR1‐mediated ERK activation [31, 52], we observed that LGG SN and fMLF shared the ability to activate MAPK signalling in CRC cells. We also verified that the proresolving response activated by LGG in CRC cells is dependent on the activation of this signalling.

In the GC model, the levels of SPMs inversely correlated with the number of proangiogenic mediators produced by cancer cells [11]. Consistently, also in the colorectal carcinoma (CRC) model, SPM production inversely correlated with the angiogenic potential of CRC cells. In particular, we observed that FPR1 activation mediated by fMLF or LGG SN significantly reduced the production of several angiogenic mediators in CRC cells. Furthermore, our experiments also demonstrated that the increased angiogenic potential of CRC cells lacking *FPR1* is due to the deficit of RvD1 and/or LXB_4_ production. On the contrary, LXA_4_, although modulated by FPR1, did not exert an antiangiogenic response in CRC cells, at least in our experimental models.

The different effects observed between RvD1/LXB_4_ and LXA_4_ suggest that probably in dependence of the mediators and on the tissue district, each SPM could exert a typical or at least predominant activity among that ascribed to SPMs (e.g. control of inflammation, limiting tissue damage, promoting resolution, attenuating fibrosis and inhibiting angiogenesis) [61].

In a mouse hepatocarcinoma cell line [62] and in a model of inflammation‐induced pathological neovascularization of the cornea [63], LXA_4_ has been reported as antiangiogenic. However, in the colon experimental model, it has been described to date that LXA_4_ could both protect against acute injury [46] and suppress CRC development [23] by specifically regulating intestinal mucosa inflammation: LXA_4_ inhibits inflammatory mediator expressions [23, 46], and reduced proinflammatory monocyte and neutrophil infiltration in tumours favouring lymphocyte activation [23, 46].

Obviously, these findings deserve a more in‐depth study in order to identify the possible different mechanisms of action (e.g. signalling pathways, receptor expression levels, cell metabolic asset) justifying the antiangiogenic properties of LXA_4_ in other tumours and not in CRC model and the differences in action compared with RvD1 and LXB_4_.

We demonstrated that the proresolving and antiangiogenic program in CRC cells could be induced by supernatants obtained by LGG cultures. Our data reinforce the idea that probiotic species contribute to an enhanced repair of mucosal wounds [32, 33] and to a protective antitumour response, which imply not only the already demonstrated antiproliferative effect [35] but also, as here presented, an antiangiogenic response on cancer cells.

These speculations are corroborated by the evidence that the properties observed for LGG are not shared with other commensal nonprobiotic bacteria, as demonstrated by our experiments on CRC cells treated with *E. coli* and by other evidence in the literature [32, 33, 64]. Furthermore, we did not observe the same effects of LGG on CRC cells neither when we used a different lactic acid probiotic strain, as *B. bifidum*. We are aware that we did not identify a specific factor/protein produced by LGG and activating FPR1; however, our experiments with *E. coli* and *B. bifidum* and previous evidence suggest that the proresolving and homeostatic functions can be ascribed only to some bacterial strains [64].

Several experimental observations in the literature suggest that SPMs or diet supplement of their precursors (ω3/6 PUFA) could integrate CRC treatment because of their ability to counteract intestinal carcinogenesis [15]. In addition, specific commensal bacteria have been identified and described as able to limit colon tumorigenesis by acting on cancer cells or on the protumorigenic inflammatory microenvironment [65]. However, to date no connection between commensal microbiota, a pattern recognition receptor sensing specific probionts and the activation of proresolving pathway has been made. We provide direct evidence of such concept, by showing that LGG can activate FPR1 sustaining the expression and function of proresolving pathways, which, in turn, suppress angiogenesis.

## 5 Conclusions

Our results consolidate the hypothesis that a correlation exists linking proresolving pathways' deficit and cancer in humans. Our data also highlight the possibility that innate immune receptors, including FPR1, could be the key regulators of the balance between microbiota recognition, inflammation regulation and neoplastic transformation. By defining the molecular mechanisms linking lipid metabolism and inflammation resolution with FPR1 in gastrointestinal (GI) tract, our data will allow the comprehension of the general mechanisms involved in tumour cell growth following their angiogenic switch and will open the possibility to identify new prognostic markers and a novel therapeutic approach for cancers of the GI tract.

## Conflict of interest

The authors declare no conflict of interest.

## Author contributions

FL participated in the design of the study, carried out experiments, drafted the manuscript, and read and approved this manuscript. MM, DS, CP, VC, GM, ES and PS carried out experiments, and read and approved this manuscript. RMM conceptualized and designed the study, analysed and interpreted the data, wrote the manuscript, and gave final approval of the manuscript and financial support. NP conceptualized and designed the study, carried out experiments, analysed and interpreted the data, wrote the manuscript and gave final approval of the manuscript.

### Peer review

The peer review history for this article is available at https://publons.com/publon/10.1002/1878‐0261.13280.

## Supporting information


**Fig. S1.** Formyl peptide receptor 1 (FPR1) expression levels in colorectal carcinoma (CRC) cells.

**Fig. S2.** *Formyl peptide receptor 1* (*FPR1*) correlation to colorectal carcinoma (CRC) patients' characteristics.

**Fig. S3.** Absence of antiangiogenic effect of LXA_4_ in colorectal carcinoma (CRC) cells.

**Fig. S4.** Antiangiogenic and proresolving effects of RvD1 and LXB4 in colorectal carcinoma (CRC) cells.

**Fig. S5.** Effects of *Lactobacillus rhamnosus* GG (LGG) supernatants LGG (SN) on specialized proresolving mediator (SPM) biosynthetic machinery and angiogenic potential of colorectal carcinoma (CRC) cells.

**Fig. S6.** Antiangiogenic effects of *Lactobacillus rhamnosus* GG (LGG) supernatants (SN) in the presence of a neutralizing anti‐GPR32 antibody.

**Fig. S7.** Annexin A1 (AnxA1) induction upon formyl peptide receptor 1 (FPR1) activation or specialized proresolving mediator (SPM) stimulation of colorectal carcinoma (CRC) cells.

**Table S1.** List of primers.

**Data S1.** Supplementary legends.

## Data Availability

All data generated or analysed during this study are included within the article or available from the corresponding author on reasonable request.
