# Supplementary material for: Probiotic Lactobacillus rhamnosus GG (LGG) restrains the angiogenic potential of colorectal carcinoma cells by activating a proresolving program via formyl peptide receptor 1
Source: Mol Oncol. 2022 Jul 20;16(16):2959–80. doi: 10.1002/1878-0261.13280 (PMC9394235; doi:10.1002/1878-0261.13280)
Supplement: Supplementary file 8 — Table S1. List of primers. [file MOL2-16-2959-s001.docx]

**Supplementary Table 1.** List of primers; F- Forward, R-Reverse in 5’-3’ direction

| *GPR32 F* | ACTATATTGTCTCCAGGCAGTG |
| --- | --- |
| *GPR32 R* | ACAGTGCGGTGGTTCAGG |
| *ChemR23 F* | CTGTCCACACCTGGGTCTTC |
| *ChemR23 R* | CCCCACAGGGTCCATTTGG |
| *BLT-1 F* | GCCCTGGAAAACGAACATGA |
| *BLT-1 R* | TTAGATGGAAGGCCCGGTG |
| *VEGF-A F* | GTGAATGCAGACCAAAGAAAG |
| *VEGF-A R* | AAACCCTGAGGGAGGCTC |
| *VEGF-B F* | TGTCCCTGGAAGAACACAGCC |
| *VEGF-B R* | GCCATGTGTCACCTTCGCA |
| *VEGF-C F* | ATGTTTTCCTCGGATGCTGGA |
| *VEGF-C R* | CATTGGCTGGGGAAGAGTTT |
| *VEGF-D F* | GTATGGACTCTCGCTCAGCAT |
| *VEGF-D R* | AGGCTCTCTTCATTGCAACAG |
| *ANG1 F* | CCTCGCTGCCATTCTGACTC |
| *ANG1 R* | ACTCTCACGACAGTTGCCATC |
| *CXCL1 F* | CACCATGGCCCGCGCTGCTCTC |
| *CXCL1 R* | GTTGGATTTGTCACTGTT |
| *ALOX15A F* | GACTTTGAGGTTTCGCTGGC |
| *ALOX15A R* | GACCACACCAGAAAATCCGG |
| *ALOX15B F* | GAGGGTACAGCCAAGGTGTC |
| *ALOX15B R* | AAAGCAGAGCCAGCCTGTAG |
| *ALOX5 F* | AGTCCTCAGGCTTCCCCAAGT |
| *ALOX5R* | CATGCCCAGGAACAGCTCGTT |
| *β-ACT F* | TGCGTGACATTAAGGAGAAG |
| *β-ACT R* | GCTCGTAGCTCTTCTCCA |
