# Supplementary material for: Probiotic Lactobacillus rhamnosus GG (LGG) restrains the angiogenic potential of colorectal carcinoma cells by activating a proresolving program via formyl peptide receptor 1
Source: Mol Oncol. 2022 Jul 20;16(16):2959–80. doi: 10.1002/1878-0261.13280 (PMC9394235; doi:10.1002/1878-0261.13280)
Supplement: Supplementary file 9 — Data S1. Supplementary legends. [file MOL2-16-2959-s009.docx]

**Supplementary Figure Legends**

**Supplementary Figure 1 Formyl peptide receptor 1 (FPR1) expression levels in colorectal carcinoma (CRC) cells**

(A) Mean fluorescence intensity in HCT116 and HT29 cells stained with an anti-FPR1 antibody or the matched isotype control. A representative experiment out of three is shown. (B) Mean fluorescence intensity in HCT116 cells silenced for FPR1 (HCT116 shFPR1, a representative clone) compared to cells transfected with non-targeting short hairpin RNAs (shCTR cells, a mass population) stained with an anti-FPR1 antibody. A representative experiment out of three is shown.

**Supplementary Figure 2 *Formyl peptide receptor 1* (*FPR1*) correlation to colorectal carcinoma (CRC) patients’ characteristics**

*Formyl peptide receptor 1* (*FPR1*) and *formyl peptide receptor 2* (*FPR2*) mRNA expression levels of 594 patients affected by colorectal adenocarcinoma stratified for overall survival status and disease-free months. Spearman factor, Pearson factor and the relative p are indicated.

**Supplementary Figure 3 Absence of anti‐angiogenic effect of LXA_4_ in colorectal carcinoma (CRC) cells**

*VEGF-A*, *VEGF-B*, *VEGF-C*, and *Ang1* mRNA fold change in HCT116 cells silenced for *formyl peptide receptor 1* (*FPR1*) (HCT116 shFPR1, three clones) compared to cells transfected with non-targeting short hairpin RNAs (shCTR cells, a mass population) following LXA_4_ (1 nM) treatment for 3 h. Data are represented as mean ± SD of five independent experiments. * p < 0.05 compared to the control (dotted line) by Student’s t test.

**Supplementary Figure 4 Anti‐angiogenic and pro‐resolving effects of RvD1 and LXB_4_ in colorectal carcinoma (CRC) cells**

(A) *VEGF-A*, *VEGF-B*, *VEGF-C*, *VEGF-D*, *Ang1*, and *CXCL1* mRNA fold change in in HCT116 cells silenced for *formyl peptide receptor 1* (*FPR1*) (HCT116 shFPR1, three clones) compared to cells transfected with non-targeting short hairpin RNAs (shCTR cells, a mass population) following treatment with LXB_4_ (1 nM) or RvD1 (1 nM) for 3 h. Data are represented as mean ± SD of five independent experiments. * p < 0.05 compared to the NT (dotted line) by Student’s t test. § p < 0.05 compared to the relative control by Student’s t test. (B) *ALOX5*, *ALOX15A*, *ALOX15B*, *GPR32*, *ChemR23*, *BLT1* mRNA fold change in HCT116 shCTR (a mass population) and shFPR1 (three clones) cells treated with LXB_4_ (1 nM) or RvD1 (1 nM) for 3 h. Data are represented as mean ± SD of five independent experiments. * p < 0.05 compared to the NT (dotted line) by Student’s t test. § p < 0.05 compared to the relative control by Student’s t test. (C) ALOX5, ALOX15A, ALOX15B, GPR32, ChemR23, BLT1 protein expression levels over control assessed by cytofluorimetric analysis in HCT116 shCTR (a mass population) and shFPR1 (three clones) cells upon treatment with RvD1 (1 nM) for 6 h. Data are represented as mean ± SD of five independent experiments. * p < 0.05 compared to the shCTR cells by Student’s t test. (D) ALOX5, ALOX15A, ALOX15B, GPR32, ChemR23, BLT1 protein expression levels over control assessed by cytofluorimetric analysis in HCT116 shCTR (a mass population) and shFPR1 (three clones) cells upon treatment with LXB4 (1 nM) for 6 h. Data are represented as mean ± SD of five independent experiments. * p < 0.05 compared to the shCTR cells by Student’s t test.

**Supplementary Figure 5 Effects of *Lactobacillus rhamnosus* GG (LGG) supernatant (SN) on specialized pro-resolving mediator (SPM) biosynthetic machinery and angiogenic potential of colorectal carcinoma (CRC) cells**

(A) *ALOX5*, *ALOX15A*, *ALOX15B*, *GPR32*, *ChemR23*, *BLT1* mRNA fold change in HT29 and HCT116 cells treated with LGG SN or the control broth (1:30 titration) for 3 h. Data are represented as mean ± SD of five independent experiments. * p < 0.05 compared to the control (dotted line) by Student’s t test. (B) *VEGF-A*, *VEGF-B*, *VEGF-C*, *VEGF-D*, *Ang1*, *CXCL1* mRNA fold change in HT29 and HCT116 cells treated with LGG SN or the control broth (1:30 titration) for 3 h. Data are represented as mean ± SD of five independent experiments. * p < 0.05 compared to the control (dotted line) by Student’s t test.

**Supplementary Figure 6 Anti‐angiogenic effects of *Lactobacillus rhamnosus* GG (LGG) supernatant (SN) in the presence of a neutralizing anti-GPR32 antibody**

*VEGF-C*, *VEGF-D*, *Ang1*, and *CXCL1* mRNA fold change in HCT116 cells treated with LGG SN - 1:30 titration or the same dilution of the culture broth for 3 h. Cells were pre-treated for 30 min with a neutralizing anti-GPR32 antibody or an isotype matched control antibody (1 μg/ml). Data are represented as mean ± SD of five independent experiments. * p < 0.05 compared to the broth by Student’s t test. § p < 0.05 compared to the relative control by Student’s t test.

**Supplementary Figure 7 Annexin A1 (AnxA1) induction upon formyl peptide receptor 1 (FPR1) activation or specialized pro-resolving mediator (SPM) stimulation of colorectal carcinoma (CRC) cells**

Mean fluorescence intensity in HCT116 and HT29 cells stained with an anti-AnxA1 antibody or the matched isotype control upon treatement with fMLF (10^-9^ M), LGG SN (1:30 titration), RvD1 (1 nM), LXB_4_ (1 nM), or LXA_4_ (1 nM). A representative experiment out of three is shown.
